# Supplementary material for: Peripheral Blood Lymphocyte Phenotype Differentiates Secondary Antibody Deficiency in Rheumatic Disease from Primary Antibody Deficiency
Source: J Clin Med. 2020 Apr 7;9(4):1049. doi: 10.3390/jcm9041049 (PMC7230453; doi:10.3390/jcm9041049)
Supplement: Supplementary file 1 [file jcm-09-01049-s001.pdf]

Supplementary file

Peripheral blood lymphocyte phenotype differentiates secondary antibody deficiency in rheumatic disease from primary antibody deficiency

Alexandra Jablonka †, Haress Etemadi †, Ignatius Ryan Adriawan, Diana Ernst, Roland Jacobs, Sabine Buyny, Torsten Witte, Reinhold Ernst Schmidt, Faranaz Atschekzei †,* and Georgios Sogkas †,*

Department of Rheumatology and Immunology, Hannover Medical School, 30625, Hannover; Jablonka.Alexandra@mh-hannover.de (A. J.); Etemadi.Haress@mh-hannover.de (H.E.); Adriawan.Ignatius@mh-hannover.de (I. A); Ernst.Diana@mh-hannover.de (E.D.); Jacobs.Roland@mh-hannover.de (R.J.); Buyny.Sabine@mh-hannover.de (S.B.); Reinhold.Ernst.Schmidt@mh-hannover.de (R. S.); Torsten.Witte@mh-hannover.de (T.W.); Atschekzei.Faranaz@mh-hannover.de (F.A.); Sogkas.Georgios@mh-hannover.de (G.S.)

* Correspondence: sogkas.georgios@mh-hannover.de; Tel.: (+49-(0)-511-532-3799) (G.S.), Atschekzei.faranaz@mh-hannover.de; Tel.: (+49-(0)-511-532-3871) (F.A.)

† These authors contributed equally

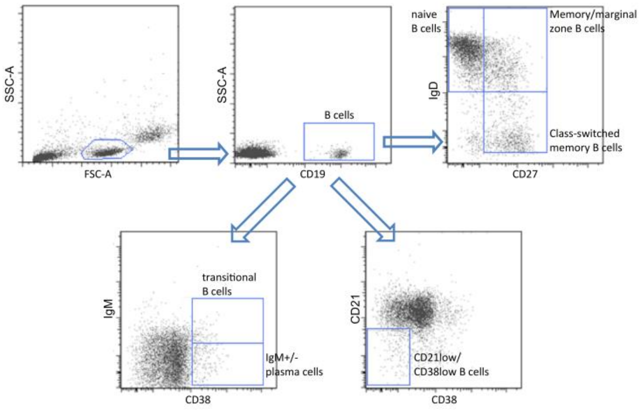

Figure S1. Strategy to measure B cell subsets. After doublet exclusion (not shown) and gating on lymphocytes based on SSC and FSC, we selected CD19⁺ B cells with subsequent gating based on IgD, IgM, CD27 and CD38 expression, as indicated.

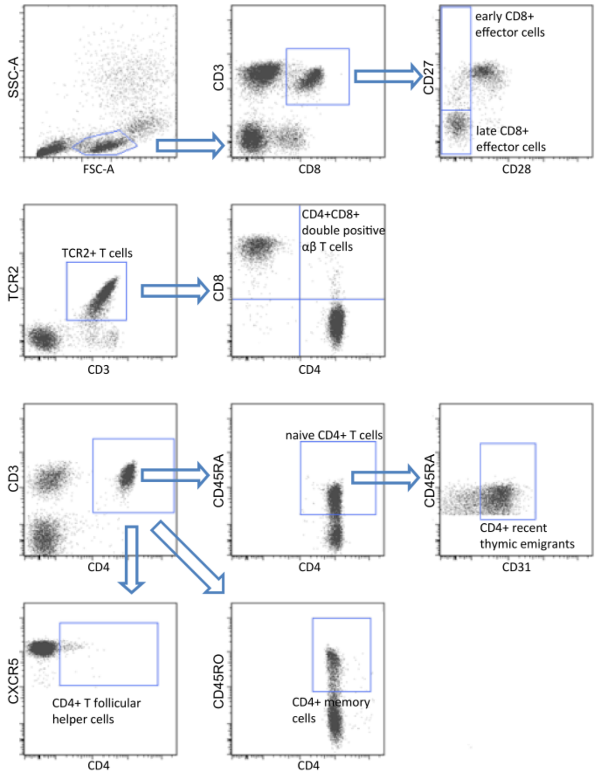

Figure S2. Strategy to measure T cell subsets. After doublet exclusion (not shown) and gating on lymphocytes based on SSC and FSC, we selected CD3+ T cells with subsequent gating based on TCR2, CD4, CD8, CD27, CD28, CXCR5, CD45RA and CD45RO expression.

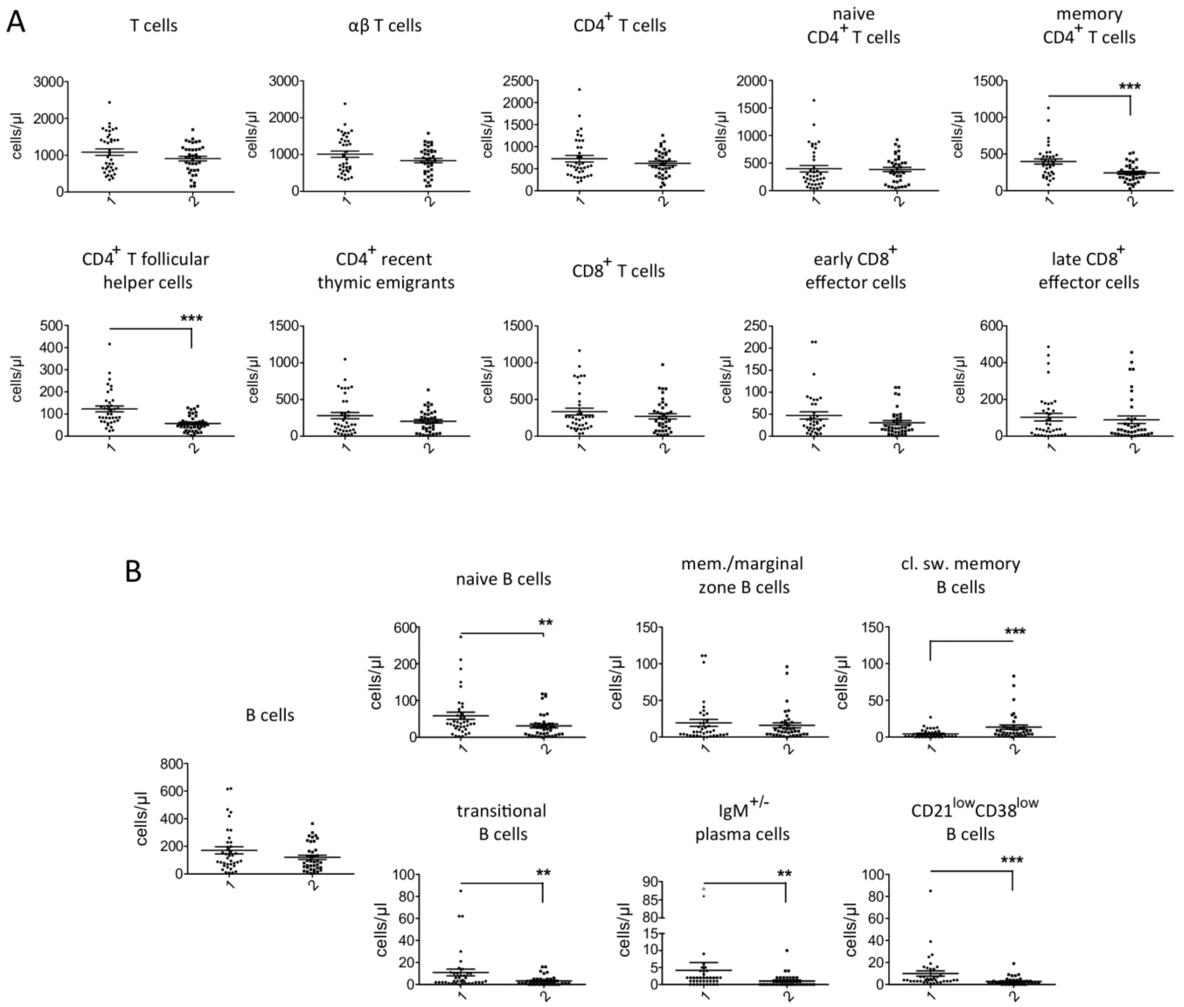

Figure S3. Comparison of absolute T cell (A) and B cell subset counts (B) in patients with primary (1, n=38) and secondary hypogammaglobulinemia (2, n=38 for T cell subsets and n=37 for B cell subsets, as a patient's (pat.nr. 37) B cells could not be differentiated due to their low number; *$p < 0.05$; **$p < 0.01$; ***$p < 0.001$).

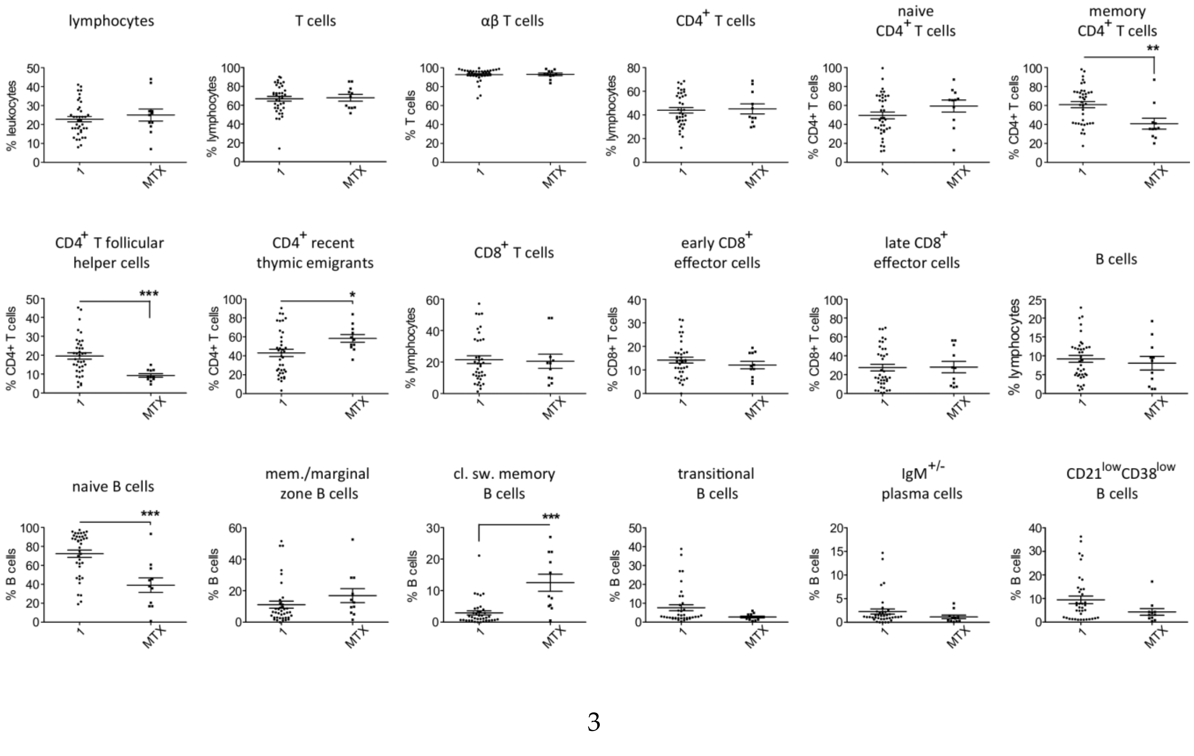

Figure S4. Comparison of B and T cell subsets in patients with primary (1, n=38) and secondary hypogammaglobulinemia due to methotrexate (MTX, n=11; *$p < 0.05$; ** $p < 0.01$; *** $p < 0.001$).

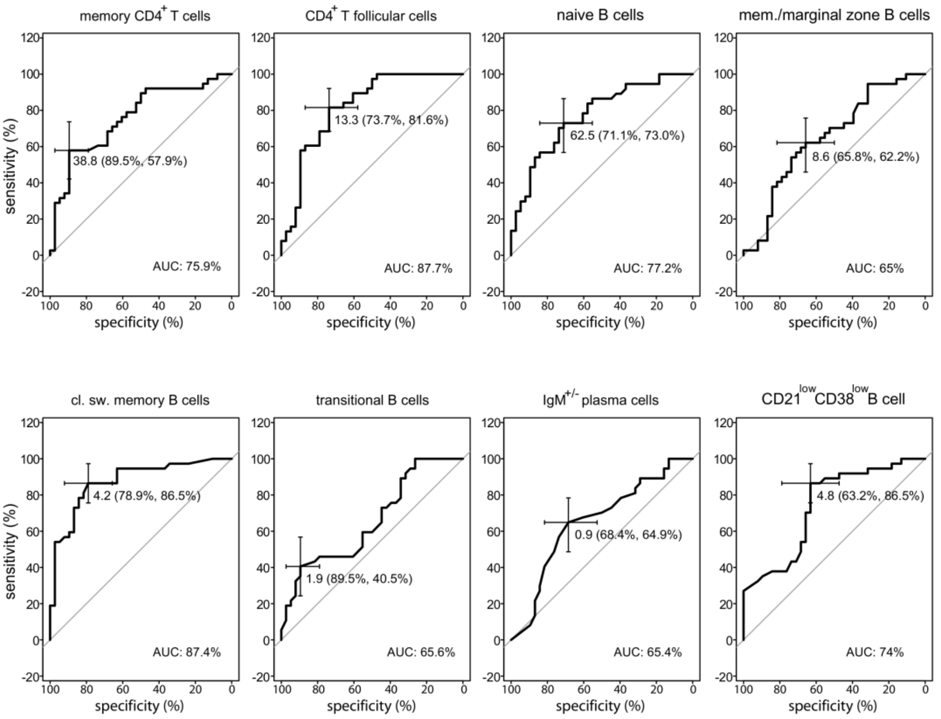

Figure S5. Receiver operating characteristic (ROC) curves and the corresponding area under the curve (AUC) values for the indicated B or T subset counts.

Table S1. Association of lymphocyte subset percentage counts with immunoglobulin values in patients with primary (1) and secondary (2) hypogammaglobulinemia.

memory CD4+ T cells (% CD4+ T cells)

	1	2
IgG	-0.295 (-56.85 - 3.7) / ns	-0.027 (-35.23 - 30.43) / ns
IgA	-0.033 (-35.8 - 29.83) / ns	0.165 (-17.27 - 46.83) / ns
IgM	-0.043 (-36.62 - 28.97) / ns	0.105 (-23.16 - 41.9) / ns

CD4+ T folicular cells (% CD4+ T cells)

	1	2
IgG	-0.172 (-47.35-16.63) / ns	-0.175 (-47.64 - 16.26) / ns
IgA	-0.043 (-36.68 - 28.92) / ns	-0.054 (-37.58 - 27.95) / ns
IgM	-0.051 (-37.36 - 28.2) / ns	0.134 (-20.37 - 44.28) / ns

mem./marginal zone B cells

	1	2
IgG	0.375 (5.28 - 62.62) / *	-0.03 (-35.97 - 30.56) / ns
IgA	0.248 (-8.79 - 53.29) / ns	0.252 (-8.8 - 54) / ns

IgM	0.464 (15.96 - 68.75) / **	0.123 (-21.89 - 43.8) / ns

naïve B cells (% B cells)

	1	2
IgG	-0.286 (-56.19 - 0.047)/ ns	-0.312 (-58.4 - 2.24) / ns
IgA	-0.262 (-54.36 - 7.3) / ns	-0.513 (-72.22 - -21.65) / **
IgM	-0.409 (-65.02 - -9.3)/ *	-0.026 (-35.57 - 30.99) / ns

cl. sw. memory B cells (% B cells)

	1	2
IgG	0.324 (-0.54 - 58.95) / *	0.395 (7.13 - 64.33) / *
IgA	0.325 (-0.4 - 59.05) / *	0.489 (18.61 - 70.67) / **
IgM	0.263 (-7.22 - 54.41) / ns	0.074 (-26.59 - 39.68) / ns

transitional B cells (% B cells)

	1	2
IgG	0.119 (-21.82 - 43.06) / ns	0.156 (-18.68 - 46.47) / ns
IgA	0.101 (-23.5 - 41.6) / ns	0.172 (-17.04 - 47.79) / ns
IgM	-0.018 (-34.49 - 31.19) / ns	-0.171 (-47.72 - 17.13) / ns

$IgM^{+/-}$ plasma cells (% B cells)

	1	2
IgG	-0.079 (-39.7 - 25.64) / ns	0.122 (-22 - 43.71) / ns
IgA	0.073 (-26.18 - 39.21) / ns	0.006 (-32.72 - 33.87) / ns
IgM	0.088 (-24.76 - 40.49) / ns	0.023 (31.15 - 35.4) / ns

CD21 ^{low}CD38^{low} B cells (% B cells)

	1	2
IgG	-0.278 (-55.57 - 0.57) / ns	0.09 (-25.09 - 41.03) / ns
IgA	-0.06 (-38.06 - 27.43) / ns	0.092 (-24.89 - 41.2) / ns
IgM	0.062 (-27.2 - 38.28) / ns	-0.008 (-34.01 - 32.58) / ns

(Spearman' s r(95% c.i.)/p-value)

Table S2. lymphocyte subset counts of patients with PID on anti-inflammatory regiments.

Pat. Nr.	Memory $CD4^+$ T cells (% $CD4^+$ T cells)	$CD4^+$ T follicular helper cells (% $CD4^+$ T cells)	Naïve B cells (% B cells)	Mem./ marginal zone B cells (% B cells)	cl. sw. memory B cells (% B cells)	Transitional B cells (% B cells)	$IgM^{+/-}$ plasma cells (% B cells)	$CD21^{low}$ B cells (% B cells)	Indication	Therapy
1	83.5	21.1	94	1.3	0.5	13.8	0.4	18	GLILD	AZA
7	74.1	23.6	90.4	8.3	0.5	2.2	1.1	4.8	PsA	MTX
14	40.6	15.1	97.4	1.8	0	35.7	1.4	1.4	GLILD	Prednisolone (5 mg)
20	75.4	13.7	85,4	4.2	2.1	2.1	0.1	6.2	IBD (Crohn's	MSZ + prednisolone

									like)	(5 mg)
21	71.7	25.3	19	1.1	0.1	1.3	1.4	13.9	GLILD	AZA
25	34.4	33.3	91.5	4.2	2.1	2.1	4.2	2.1	perSpA	MTX

AZA, azathioprine; GLILD, *Granulomatous–lymphocytic interstitial lung disease;* IBD, inflammatory bowel disease; MSZ, mesalazine; MTX, methotrexate; pat. nr., patient number; perPsA, periphere spondyloarthritis; PsA, psoriasis arthritis
